# The clinical trial landscape of bispecific antibodies in non-small cell lung cancer: mechanistic trends and developmental strategies

**DOI:** 10.3389/fphar.2026.1901850

**Published:** 2026-09-03

**Authors:** Zunyuan Li, Xiyu Wang, Qin Shi, Yuhong Guo, Guirong Dong, Zhidan Liu

**Affiliations:** 1 Baoshan Hospital, Shanghai University of Traditional Chinese Medicine (Shanghai Baoshan Hospital of Integrated Traditional Chinese and Western Medicine), Shanghai, China; 2 Yueyang Hospital of Integrated Traditional Chinese and Western Medicine, Shanghai University of Traditional Chinese Medicine, Shanghai, China

**Keywords:** bispecific antibodies, clinical trials, immune checkpoint, immunotherapy, non-small cell lung cancer, T-cell engagers, tumor microenvironment

## Abstract

**Background:**

Non-small cell lung cancer (NSCLC) management remains constrained by acquired resistance to targeted therapies and refractoriness to immune checkpoint inhibitors (ICIs), the latter driven largely by the immunosuppressive tumor microenvironment (TME). Bispecific antibodies (bsAbs) represent a promising modality capable of circumventing these therapeutic hurdles through proximity-driven dual-targeting mechanisms that co-block bypass signaling pathways and reverse immunosuppression. This study systematically analyzes the clinical trial landscape and developmental trajectory of bsAbs in NSCLC.

**Methods:**

We systematically queried the Trialtrove database for interventional clinical trials evaluating bsAbs in NSCLC with an actual or anticipated study start date on or before 8 February 2026. Following the application of predefined inclusion and exclusion criteria, 374 trials were identified. Data concerning trial phase, mechanistic targets, sponsor typology, and geographic distribution were extracted and analyzed using a mechanism-based classification framework.

**Results:**

The clinical pipeline has expanded rapidly, with 78.9% (295/374) of included trials initiated since 2021, predominantly led by industry sponsors (82.9%). The landscape is characterized by a predominance of early-phase studies, with Phase I and I/II trials comprising 62.6% (234/374) of the cohort, compared with 9.9% (37/374) advancing to late-stage pivotal validation. Dual-signaling inhibitors and checkpoint-TME bsAbs constitute the majority of late-stage investigations, whereas T-cell engagers (TCEs) form a rapidly expanding early-phase cohort. Geographically, the United States and China collectively participate in 94% of trials with available location data, but their strategic priorities diverge. The US pipeline emphasizes early-stage, high-risk mechanistic exploration (74% in Phase I or I/II), whereas trials conducted in mainland China are concentrated in late-stage validation of established target combinations.

**Conclusion:**

The clinical development of bsAbs in NSCLC has shifted decisively toward rationally engineered constructs designed to address specific resistance mechanisms. The global landscape reflects complementary regional strategies that balance high-risk mechanistic innovation with accelerated clinical execution. The integration of these precision-engineered modalities into standard care has the potential to reshape the therapeutic paradigm for advanced NSCLC.

## 1 Introduction

Non-small cell lung cancer (NSCLC) remains the leading cause of cancer-related mortality worldwide (Bray et al., 2024). Targeted therapies and immune checkpoint inhibitors (ICIs) have transformed the management of NSCLC (Sharma et al., 2017). However, durable clinical benefit remains limited by acquired resistance to targeted therapies, including bypass pathway activation (Wu and Shih, 2018). Immune checkpoint inhibition is similarly constrained by primary or acquired resistance, often involving an immunosuppressive tumor microenvironment (TME) (Sharma et al., 2017). Overcoming these entrenched mechanisms demands therapeutic modalities that extend beyond the functional constraints of conventional monoclonal antibodies. Bispecific antibodies (bsAbs) have emerged as a compelling therapeutic class in this context. Engineered to simultaneously engage two distinct epitopes, bsAbs enable proximity-driven biological effects—including obligate immune-cell cross-linking and coordinated pathway co-blockade—that are not achievable with conventional monospecific agents (Klein et al., 2024). These properties position bsAbs as uniquely suited to reprogram the TME and counteract complex resistance networks.

Driven by this biological rationale, the clinical pipeline for NSCLC bsAbs has expanded considerably. Existing reviews provide valuable perspectives but differ in scope from the present study, focusing on bsAb structural formats and antibody engineering, the immunological basis of dual targeting, or the clinical performance of individual agents (Herrera et al., 2024; Guidi et al., 2025; Zhou et al., 2025). What has not been established is a systematic, trial-level description of the pipeline as a whole. Such an account would show how development activity is distributed across mechanistic classes, and which classes have advanced to pivotal validation rather than remaining confined to early exploration. It would also show how development effort is divided between industry and academic sponsors, and how regional strategies diverge.

This gap is consequential. Decisions on target prioritization, trial design, and portfolio positioning are made against the state of the whole pipeline rather than that of any single agent, and no such account has previously been assembled for NSCLC. To address this, we conducted a systematic analysis of 374 bsAb clinical trials in NSCLC. By delineating the temporal evolution of developmental targets, uncovering sponsor-driven and geographic strategic divergences, and examining structural shifts in trial design, this study elucidates the translational logic underpinning the global bsAb pipeline. This analysis provides a data-driven foundation to inform future target prioritization and clinical trial design in the evolving NSCLC therapeutic landscape.

## 2 Methods

### 2.1 Search strategy and data sources

To comprehensively map the clinical development landscape of bsAbs in NSCLC, data were systematically acquired from the Trialtrove database (Citeline, Pharma Intelligence). Trialtrove is a globally recognized, continuously updated repository that aggregates clinical trial data from over 30,000 sources, including ClinicalTrials.gov, the European Union Clinical Trials Register (EU-CTR), and the Chinese Clinical Trial Registry (ChiCTR).

A structured search strategy was employed using the database’s drug and disease ontologies. Trials were retrieved if the primary tested drug was classified under either of two Drug Type categories: “Biological > Protein > Antibody > Bispecific antibody” or “Biological > Protein > Antibody > Cell engager, bispecific.” These were combined with a Disease field of “Oncology: Lung, Non-Small Cell” and an actual or anticipated study start date on or before 8 February 2026, which served as the data cutoff. No filters were applied to trial phase or study type at the retrieval stage; the resulting 470 records therefore included a small number of observational and retrospective studies, which were removed during manual screening (Section 2.2).

**FIGURE 1:**
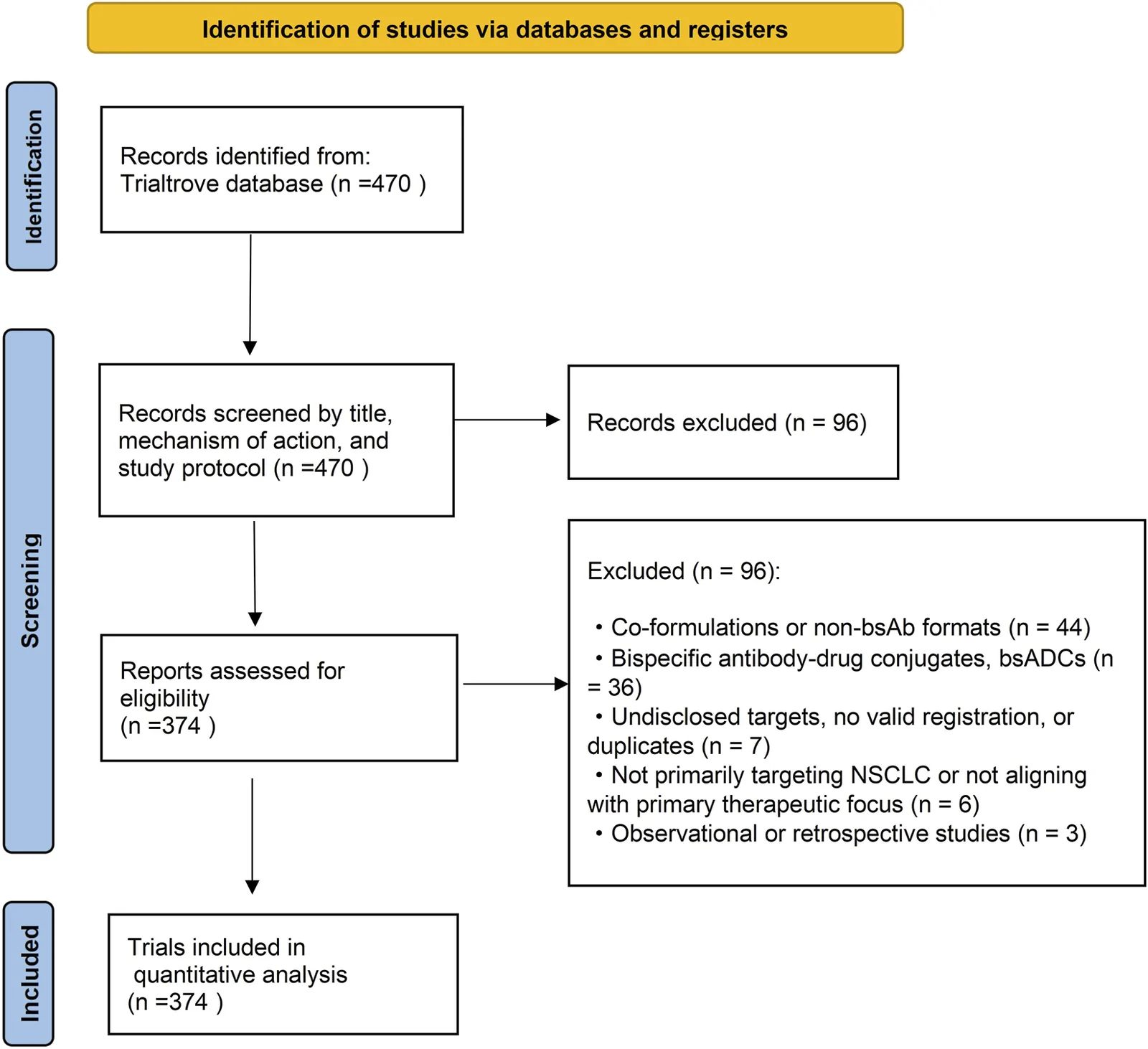
PRISMA flow diagram of trial identification and selection. The screening unit was the database record, whereas the final unit of analysis was the individual clinical trial. A total of 470 records were identified from the Trialtrove database; after applying the predefined exclusion criteria, 374 clinical trials were included in the quantitative analysis. PRISMA, Preferred Reporting Items for Systematic Reviews and Meta-Analyses (Page et al., 2021).

The complete search strategy—including the Trialtrove search fields, hierarchical ontology terms, Boolean relationships, date restriction, exact exported query, and total number of records retrieved—is provided in Supplementary Table S1. The screening workflow and the numbers excluded and retained at each stage are shown in Figure 1. Extracted variables and their handling, the sponsor-type precedence rule, and the mechanistic classification codebook are provided in Supplementary Table S2.

The research question was structured according to the PICOS framework as follows: Population (P): interventional clinical trials evaluating bispecific antibodies in patients with NSCLC; Intervention (I): bispecific antibody-based therapeutic agents; Comparator (C): not applicable, as this study aimed to characterize the overall clinical trial landscape rather than compare therapeutic efficacy; Outcomes (O): trial phase distribution, mechanistic target classification, primary sponsor typology, and geographic distribution patterns; Study design (S): a systematic analysis of clinical trial registration data retrieved from the Trialtrove database.

### 2.2 Trial selection and data extraction

To ensure data accuracy and minimize false-positive inclusions, the raw dataset underwent a rigorous screening process in accordance with the PRISMA 2020 guidelines (Page et al., 2021) (Figure 1, PRISMA flow diagram).

Two independent investigators (ZL and XW) systematically reviewed the titles, mechanisms of action, and study protocols of all initially retrieved records. Records were manually excluded if they met any of the following criteria: (1) observational studies, retrospective analyses, expanded access programs, or expanded cohort frameworks without independent trial registration; (2) co-formulations of distinct monoclonal antibodies or other non-bispecific antibody formats misclassified by the database algorithm; (3) bispecific antibody-drug conjugates (bsADCs); (4) trials with undisclosed molecular targets, lacking valid registration numbers, or duplicate records; (5) trials not primarily targeting NSCLC or not aligning with the primary therapeutic focus of this study. Discrepancies were resolved through consensus or via arbitration by a third senior investigator (ZL).

For each eligible trial, data were extracted and compiled into a proprietary database. The extracted variables included: trial identifier, trial initiation year, primary sponsor type, trial phase, operational status, study locations, investigational drugs, and specific molecular targets. Geographic distribution was analyzed based on registered study site countries. As multi-regional clinical trials (MRCTs) may enroll participants across multiple countries, a single trial could contribute to the count of more than one country. Trials without available geographic information were excluded from the geographic analysis. The extracted molecular targets served as the primary variable for our subsequent mechanistic classification. Furthermore, a qualitative review of the study protocols was conducted to identify specific clinical trial designs (e.g., seamless adaptive designs) and combination therapy strategies. This protocol-level analysis provided the contextual data required for our subsequent landscape evaluation.

Potential duplicates were identified by cross-checking trial identifiers, registration numbers, protocol information, sponsors, and investigational agents, and only one record was retained for each confirmed trial. Trials evaluating the same investigational agent were then grouped at the asset level after standardization of drug names and development codes; analyses reported at the asset level are identified as such, and all others use the 374-trial denominator. Trial phase, initiation year, operational status, and sponsor type were available for all included trials, and missing values were not imputed. Where more than one sponsor type was listed, a single category was assigned using a fixed precedence rule (Supplementary Table S2). Twelve trials lacked study-site information and were excluded from the geographic analyses only (n = 362). Where more than one termination reason was recorded for a trial, a single category was assigned by a predefined precedence rule, so that termination categories are mutually exclusive.

### 2.3 Mechanistic target classification and milestone identification

Currently, the classification of bispecific antibodies (bsAbs) encompasses various structural and functional dimensions. To closely align with the specific therapeutic landscape of NSCLC, this study employed a mechanism-driven taxonomy. We adapted core mechanistic frameworks from recent landmark reviews (Dahlén et al., 2018; Rader, 2020; Herrera et al., 2024; Klein et al., 2024; Guidi et al., 2025) and categorized the investigational bsAbs based on their biological rationales and pipeline distribution frequencies within our dataset. Classification criteria were defined *a priori*, before data extraction, and were recorded in a written codebook that was applied unchanged throughout (Supplementary Table S2). Bispecific antibody-drug conjugates (bsADCs) were strictly excluded from this core classification to independently evaluate the intrinsic immunomodulatory and signaling properties of the unconjugated bispecific formats.

Based on these criteria, the included bsAb pipeline was fundamentally categorized into non-immunomodulatory constructs, immunomodulatory constructs, and other exploratory modalities. These were further stratified into the following functional classes.

#### 2.3.1 Non-immunomodulatory bsAbs

##### 2.3.1.1 Dual signaling inhibitors

Molecules designed to simultaneously antagonize two independent oncogenic signaling cascades or bypass activation pathways (e.g., EGFR × c-MET, EGFR × HER3). In the clinical context of NSCLC, the primary objective of these molecules is to circumvent or overcome acquired resistance to tyrosine kinase inhibitors (TKIs) (Herrera et al., 2024; Klein et al., 2024).

#### 2.3.2 Immunomodulatory bsAbs

##### 2.3.2.1 T-cell engagers (TCEs)

Synthetic macromolecules engineered to physically bridge tumor-associated antigens (TAAs) with T-cell surface activating receptors (predominantly CD3). This mechanism redirects T cells to the tumor microenvironment in an MHC-independent manner, thereby inducing potent, targeted cytotoxicity (Klein et al., 2024; Zhou et al., 2025).

##### 2.3.2.2 Dual immune checkpoint inhibitors (dual CPIs)

Functioning as dual immunomodulators, these molecules concurrently block two distinct immunosuppressive checkpoints (e.g., PD-1 × CTLA-4, PD-L1 × LAG-3). Their mechanism aims to synergistically reverse deep CD8^+^ T-cell exhaustion by abrogating nonredundant inhibitory signals (Joller et al., 2024; Klein et al., 2024).

##### 2.3.2.3 Checkpoint-TME factor dual-targeting bsAbs (checkpoint-TME bsAbs)

Molecules that synchronously target a classical immune checkpoint (e.g., PD-1 or PD-L1) and a non-checkpoint modulator of the tumor microenvironment (TME), comprising soluble suppressive factors (e.g., VEGF, TGF-β), immunostimulatory cytokines (e.g., IL-2, IL-15), and innate immune checkpoints (e.g., CD47, CD39). As a prominent focal point in current lung cancer research, this design logic seeks to ameliorate abnormal angiogenesis or stromal hyperplasia, thereby reversing immune exclusion and remodeling immunologically “cold” tumors into “hot” phenotypes (Mpekris et al., 2020; Klein et al., 2024).

##### 2.3.2.4 Co-stimulatory bsAbs

Molecules that incorporate an agonistic T-cell co-stimulatory receptor (e.g., 4-1BB, OX40, ICOS, CD27, CD28) as one arm, paired either with an immune checkpoint (e.g., PD-L1 × 4-1BB) or with a tumor-associated antigen (e.g., CEA × 4-1BB). This mechanism relies heavily on target crosslinking within the TME to achieve conditional, tumor-localized immune agonism, effectively circumventing the severe systemic toxicity barriers historically associated with conventional monoclonal agonists (Klein et al., 2024; Muik et al., 2022).

##### 2.3.2.5 Tumor-targeted immunomodulators

Bispecific constructs that bind a TAA alongside an immune checkpoint or immunomodulatory receptor on innate or adaptive immune cells (e.g., HER2 × PD-1, CLDN18.2 × CD47, EpCAM × CD40). Receptors in this category act primarily on antigen-presenting or innate immune cells (e.g., CD40, CD47), in distinction to the T-cell co-stimulatory receptors defining the preceding class. This format is designed to restrict immune checkpoint blockade or immune cell activation specifically to the tumor site, thereby mitigating systemic toxicity (Klein et al., 2024; Zhou et al., 2025).

#### 2.3.3 Other exploratory modalities

##### 2.3.3.1 Other targets

To ensure statistical completeness, a minor subset of bsAbs involving unconventional mechanisms with low clinical frequency in NSCLC were consolidated into this auxiliary category. This category includes innate or unconventional cell engagers (e.g., EGFR × CD16A, EGFR × Vγ9Vδ2).

Mechanistic classification was performed at the unique-asset level, after grouping trials evaluating the same investigational bsAb; all trials of a given asset therefore share its classification. Assignment was determined by the identity of the engaged targets rather than by narrative descriptions of therapeutic intent. Where a construct could satisfy more than one category, a fixed precedence order was applied: engagement of a T-cell activating receptor (CD3) took precedence over all other features; engagement of a T-cell co-stimulatory receptor took precedence over checkpoint or tumor-antigen features; and among the remaining constructs, assignment followed the composition of the target pair. Because the rule operates on target identity rather than on investigator judgment, assignment is reproducible from the target pair itself.

Classification decisions were independently reviewed by two investigators (ZL and XW). Disagreements were resolved by discussion and, where necessary, by a third senior investigator (ZL). Assets for which the available information was insufficient to identify both targets were retained and reported within the “other targets” category rather than being forced into a mechanistic class.

To objectively highlight key developmental milestones, representative bsAb candidates (Table 1) were systematically identified from the deduplicated dataset. For each unique molecular target combination, the most clinically advanced candidate—defined by the highest trial phase and, where applicable, the earliest trial initiation year—was selected. For mechanistic classes lacking Phase III representation in NSCLC, the single most advanced asset was included to ensure comprehensive mechanistic coverage.

**TABLE 1:** Representative bsAb candidates and key clinical milestones in NSCLC.

Mechanistic class	Drug candidate	Molecular target	Sponsor	Country	Highest phase	Key trial(s)	Pivotal milestone and therapeutic rationale
Dual signaling inhibitors	Amivantamab (JNJ-61186372)	EGFR × c-MET	Johnson and Johnson	USA	Phase III	MARIPOSA	FDA-approved agent: Superior PFS versus osimertinib in 1L EGFR-mutated advanced NSCLC (MARIPOSA).
​	Izalontamab (SIB001)	EGFR × HER3	Systimmune	China	Phase III	NCT05943795	Resistance-driven target: Addresses HER3-mediated bypass signaling in patients progressing on standard EGFR-TKIs.
Dual CPIs	Cadonilimab (AK104)	PD-1 × CTLA-4	Akeso	China	Phase III	AK104-303	Dual checkpoint blockade: Advancing into 1L NSCLC in combination with chemotherapy.
Checkpoint-TME bsAbs	Ivonescimab (AK112)	PD-1 × VEGF	Akeso	China	Phase III	HARMONi-2	Head-to-head validation: Significantly improved PFS over pembrolizumab monotherapy in 1L PD-L1-positive NSCLC (HARMONi-2).
​	SHR-1701	PD-L1 × TGF-β	Hengrui	China	Phase III	NCT05812066	Reversing immune exclusion: Designed to neutralize TGF-β-mediated stromal suppression, targeting both ICI-naive and ICI-refractory phenotypes.
​	IBI363	PD-1 × IL-2α-bias	Innovent	China	Phase III	NCT07217301 (MarsLight-11)	First-in-class IL-2 engager: Encouraging early efficacy in PD-1-refractory squamous NSCLC; FDA Fast track and NMPA breakthrough therapy designations granted.
Co-stimulatory bsAbs	Acasunlimab (GEN1046)	PD-L1 × 4-1BB	Genmab/BioNTech	Denmark	Phase III	NCT06635824 (ABBIL1TY NSCLC-06)	Conditional co-stimulation: Durable post-ICI responses via tumor-localized 4-1BB agonism with manageable hepatotoxicity.
TCEs	Cibisatamab (RO6958688)	CEA × CD3	Roche	Switzerland	Phase I/II	NCT02650713	2:1 format design: Dual CEA-binding domains to enhance tumor avidity; explored in combination with anti-PD-L1 therapy.
Tumor-targeted immunomodulators	IMM2902	HER2 × CD47	ImmuneOnco	China	Phase I/II	NCT05212517	Innate checkpoint modulation: CD47 blockade designed to avoid the hematological toxicities of monospecific CD47 antibodies.

Abbreviations: 1L, first-line; 4-1BB, tumor necrosis factor receptor superfamily member 9; bsAb, bispecific antibody; c-MET, mesenchymal-epithelial transition factor; CD3, cluster of differentiation 3; CD47, cluster of differentiation 47; CEA, carcinoembryonic antigen; CPI, checkpoint inhibitor; CTLA-4, cytotoxic T-lymphocyte-associated protein 4; EGFR, epidermal growth factor receptor; FDA, food and drug administration; HER2, human epidermal growth factor receptor 2; HER3, human epidermal growth factor receptor 3; ICI, immune checkpoint inhibitor; IL-2, interleukin-2; NSCLC, non-small cell lung cancer; PD-1, programmed cell death protein 1; PD-L1, programmed death-ligand 1; PFS, progression-free survival; TCE, T-cell engager; TGF-β, transforming growth factor beta; TKI, tyrosine kinase inhibitor; TME, tumor microenvironment; VEGF, vascular endothelial growth factor.

### 2.4 Statistical analysis

Descriptive statistics were used to summarize the clinical trial landscape. Categorical variables, including trial phase, primary sponsor type, molecular targets, target classification, and regional distribution, were reported as frequencies and percentages. Annual trends in trial initiation were calculated from registered start dates. Cross-tabulation was performed to evaluate the distribution of clinical phases across sponsor categories. All data processing, standardization, and visualization were performed using Python (Google Colaboratory).

Categorical distributions were compared using Pearson’s chi-square test, with Yates’ continuity correction applied to 2 × 2 tables, and Fisher’s exact test for the targeted comparison between T-cell engagers and all other mechanistic classes. Where the assumptions of the chi-square approximation were not met, clinical phase was collapsed for inferential testing into early development and Phase II or later, and P values for tables with sparse expected cell counts were estimated by a Monte Carlo procedure; the test applied to each comparison is specified in Supplementary Table S3. Country-level descriptive analyses used participation-based counts, whereas inferential comparisons were restricted to mutually exclusive US-only and mainland-China-only subsets. Effect sizes were reported as Cramér’s V, or as odds ratios with 95% confidence intervals for 2 × 2 comparisons. All tests were two-sided, with P < 0.05 considered significant. Analyses were performed in Python 3 using SciPy.

## 3 Results

### 3.1 Overview of the clinical trial landscape

**FIGURE 2:**
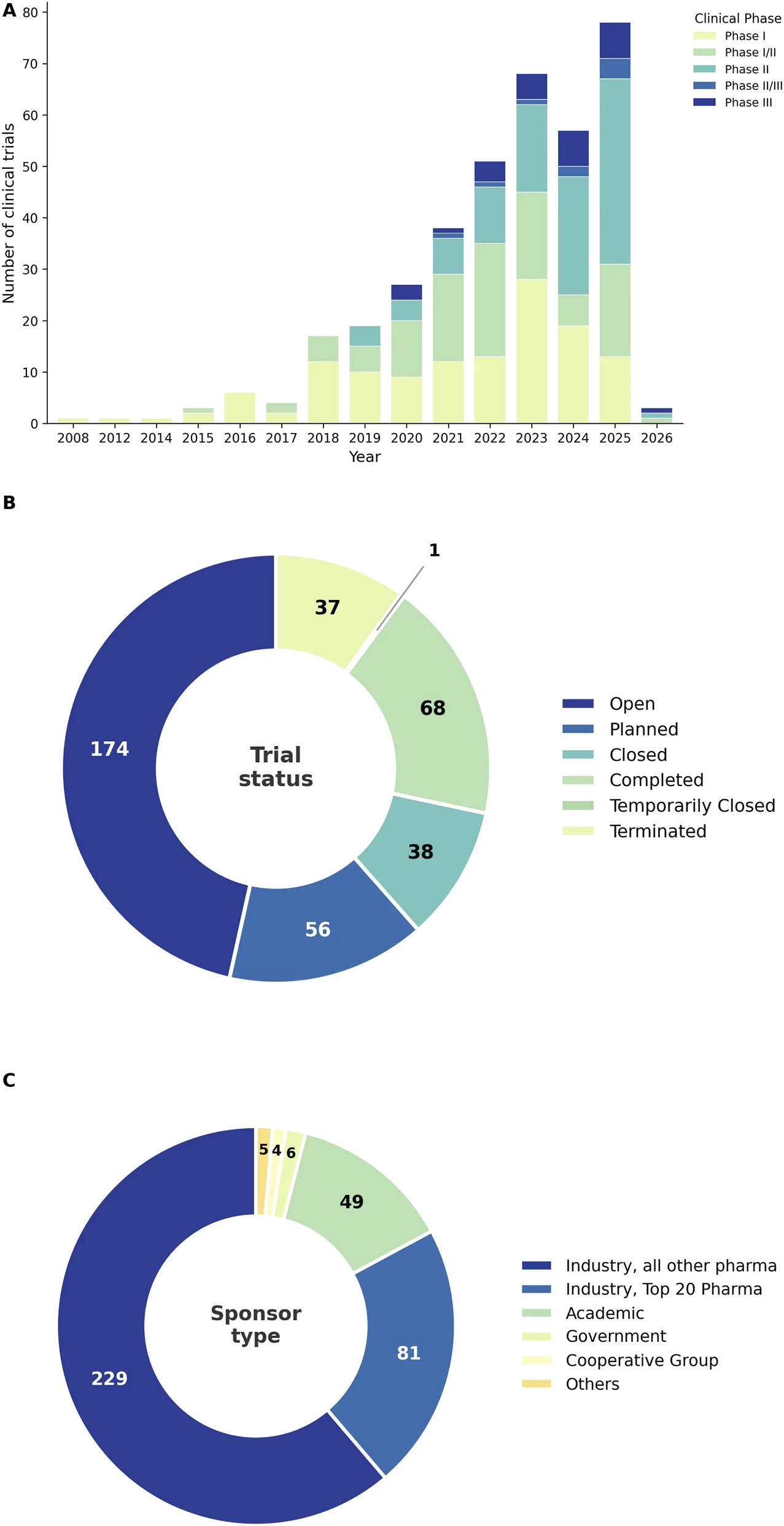
Overview of the bsAb clinical trial landscape in NSCLC. The unit of analysis in all panels was the individual clinical trial (N = 374). **(A)** Annual distribution of newly initiated trials by clinical phase, 2008–2026; data for 2026 are incomplete because the search cutoff was 8 February 2026. **(B)** Distribution of trial operational status at data extraction. **(C)** Distribution of primary sponsor type; when more than one sponsor type was listed, a single category was assigned using the precedence rule in Supplementary Table S2.

As of February 2026, 374 clinical trials investigating bsAbs in NSCLC were included in the final analysis. The clinical pipeline has expanded rapidly, with 78.9% (295/374) of trials initiated since 2021 (Figure 2A). Despite this growth, the landscape remains concentrated in early stages: Phase I and Phase I/II trials constituted 62.6% (234/374) of the cohort, in contrast to late-stage pivotal investigations (Phase II/III and Phase III), which accounted for 9.9% (37/374). The landscape is dynamic and predominantly industry-driven, with 61.5% of trials currently recruiting or planned (Figure 2B) and 82.9% (310/374) sponsored by pharmaceutical companies (Figure 2C).

Of the 374 trials, 37 (9.9%) were terminated. TCEs exhibited the highest class-specific termination rate (14/52, 26.9%), followed by co-stimulatory bsAbs (4/35, 11.4%) and dual CPIs (9/98, 9.2%), while checkpoint-TME bsAbs demonstrated the lowest rate (3/109, 2.8%). The majority of terminations were attributed to business or strategic decisions. Of the 37 terminated trials, 3 were coded as lack of efficacy, 1 as safety concerns, and 2 as poor enrollment; the remainder were coded as business or strategic decisions or fell into other or unknown categories.

Termination rates differed across the six defined mechanistic classes (n = 363; the heterogeneous “other targets” category was excluded; χ^2^ = 25.78, df = 5, Monte Carlo P < 0.001; Cramér’s V = 0.27). Across the full cohort, T-cell engagers showed a higher termination rate than all other classes combined (26.9% [14/52] vs. 7.1% [23/322]; OR 4.79, 95% CI 2.27–10.09; Fisher’s exact P < 0.001; Supplementary Table S3).

### 3.2 Evolution of mechanistic targets and key clinical candidates

**FIGURE 3:**
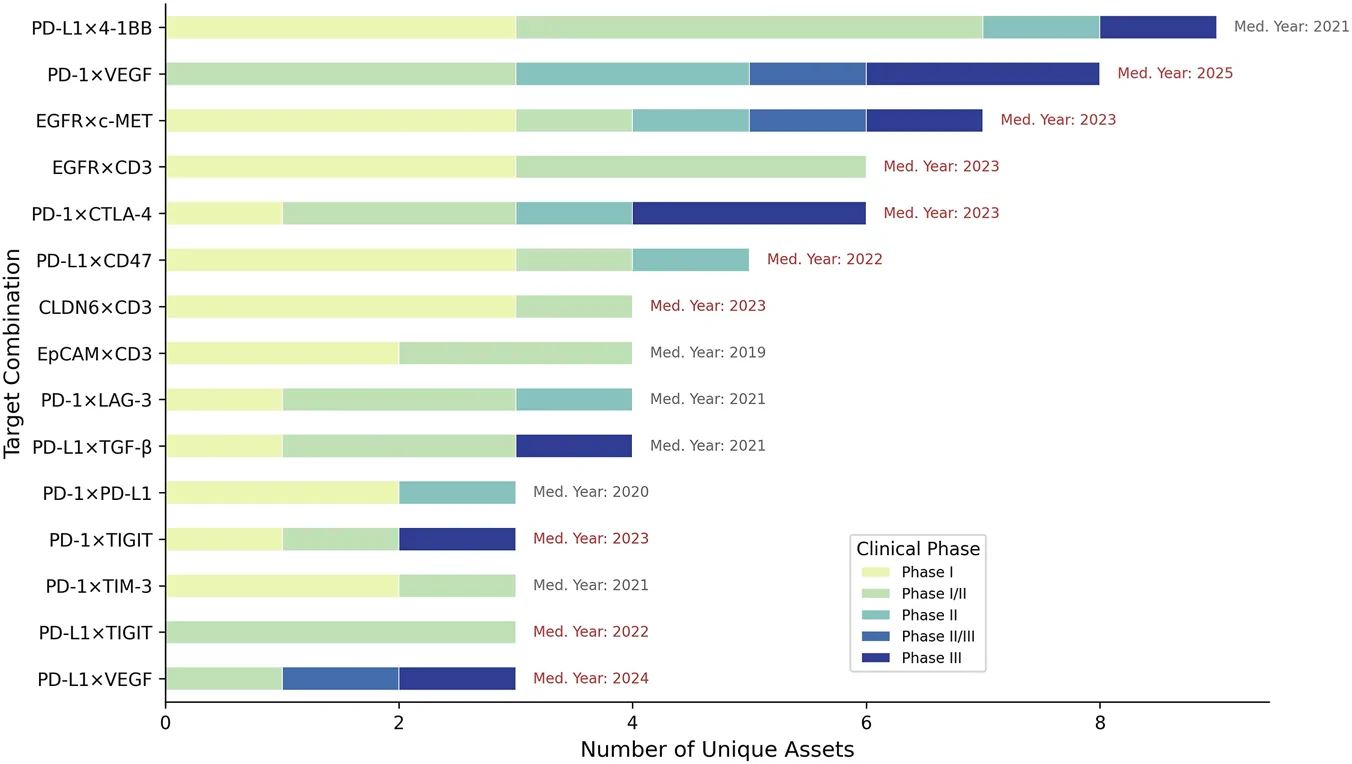
Top 15 target combinations ranked by the number of unique clinical-stage bsAb assets. The unit of analysis was the deduplicated bsAb asset (n = 170), not the clinical trial. Horizontal bars show the number of unique assets per target combination, stratified by the highest clinical phase reached by each asset. Med. Year denotes the median initiation year of trials evaluating assets in that target combination. Red labels indicate combinations with a median initiation year of 2022 or later.

After adjusting for molecules investigated across multiple trials, deduplication identified 170 unique bsAb assets under clinical evaluation for NSCLC. Analysis of the top 15 most frequently targeted bispecific combinations reveals a concentrated, mechanism-driven landscape dominated by immunomodulatory constructs (Figure 3). Checkpoint-TME dual-targeting strategies and TCEs exhibit notable pipeline diversity, collectively accounting for the largest proportion of unique assets. Among the top 15 most frequently targeted combinations, dual signaling blockade is exclusively represented by the EGFR × c-MET axis.

**FIGURE 4:**
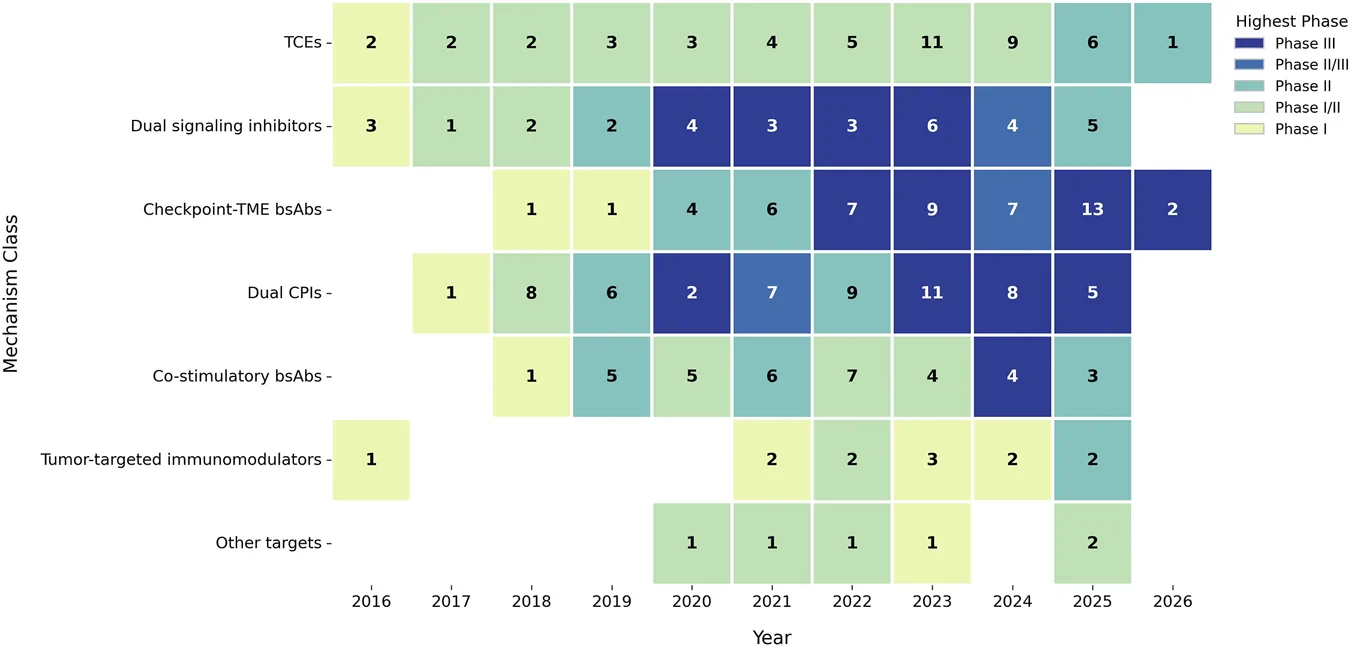
Temporal evolution of pipeline breadth and clinical maturity by mechanism class. The unit of analysis was the unique bsAb asset within each mechanism class–year cell. For an asset represented by more than one trial in the same class and year, only its highest phase was retained. Cell values indicate the number of unique assets represented in that year, whereas cell color indicates the highest clinical phase reached within the class–year cell. An asset could contribute to more than 1 year if trials evaluating that asset were initiated in different years. Data for 2026 are incomplete because the search cutoff was 8 February 2026.

Integration of trial initiation years with clinical phase data delineates distinct developmental trajectories across mechanistic classes (Figure 4). During the foundational period (pre-2021), the pipeline was primarily characterized by early exploration of dual signaling inhibitors and dual CPIs. Since 2021, the overall pipeline has expanded substantially across all mechanistic categories, with these early entrants steadily progressing toward late-stage investigations.

Most notably, checkpoint-TME bsAbs have exhibited rapid growth since 2021. Despite their recent emergence, this class has matured into the largest pipeline segment, with a substantial proportion of trials advancing to Phase III. In contrast, while TCEs have maintained a continuous presence since the earliest stages of bsAb development, their clinical maturity remains low, with the vast majority of assets concentrated in early-phase dose-escalation studies.

These developmental trajectories align with landmark clinical milestones achieved by leading candidates within these dominant classes (Table 1).

### 3.3 Geographical disparities and divergent clinical trajectories

**FIGURE 5:**
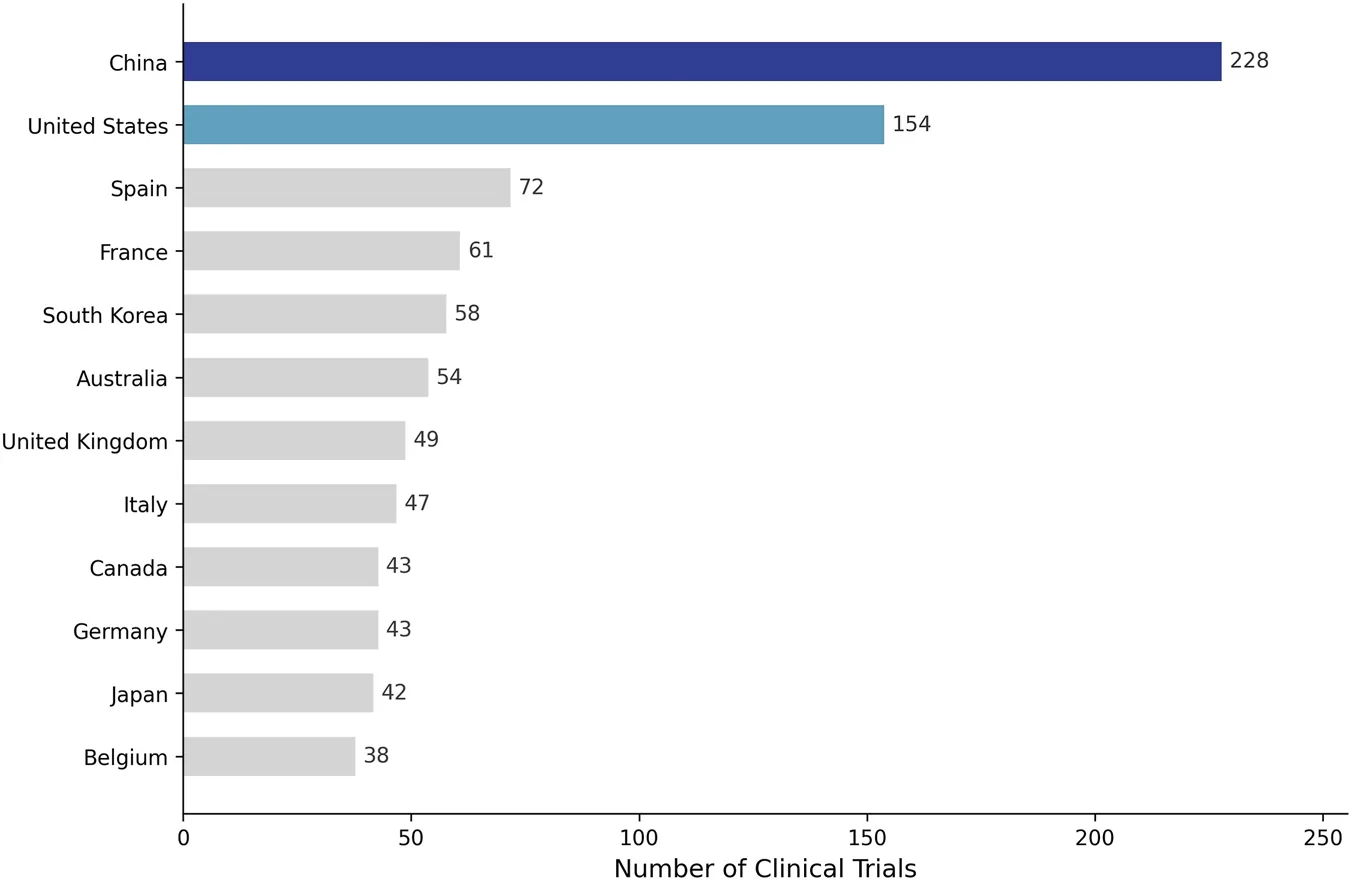
Top 12 countries by clinical trial participation. The unit of analysis was the country–trial participation. Of the 374 included trials, 362 with available geographic information were analyzed. Because a multi-regional trial could involve sites in several countries, one trial could contribute to the count of every participating country; country totals were therefore not mutually exclusive and did not sum to 362. Mainland China excluded Hong Kong SAR and Taiwan, which were counted separately.

Of the 374 included trials, 362 (96.8%) had identifiable geographic information. Geographic analysis of the 362 trials with available location data reveals a highly concentrated landscape, with the United States and China collectively participating in 94.2% (341/362) of clinical trials (Figure 5). Other traditional pharmaceutical hubs, including Europe and Japan, represent a comparatively minor fraction of the current developmental landscape.

Because multi-regional trials contributed to the count of every participating country, country-level descriptive totals were not mutually exclusive. A trial involving sites in both the United States and mainland China contributed to both participation-based groups. Inferential comparisons therefore used mutually exclusive US-only and mainland-China-only subsets, as defined in Supplementary Table S3.

**FIGURE 6:**
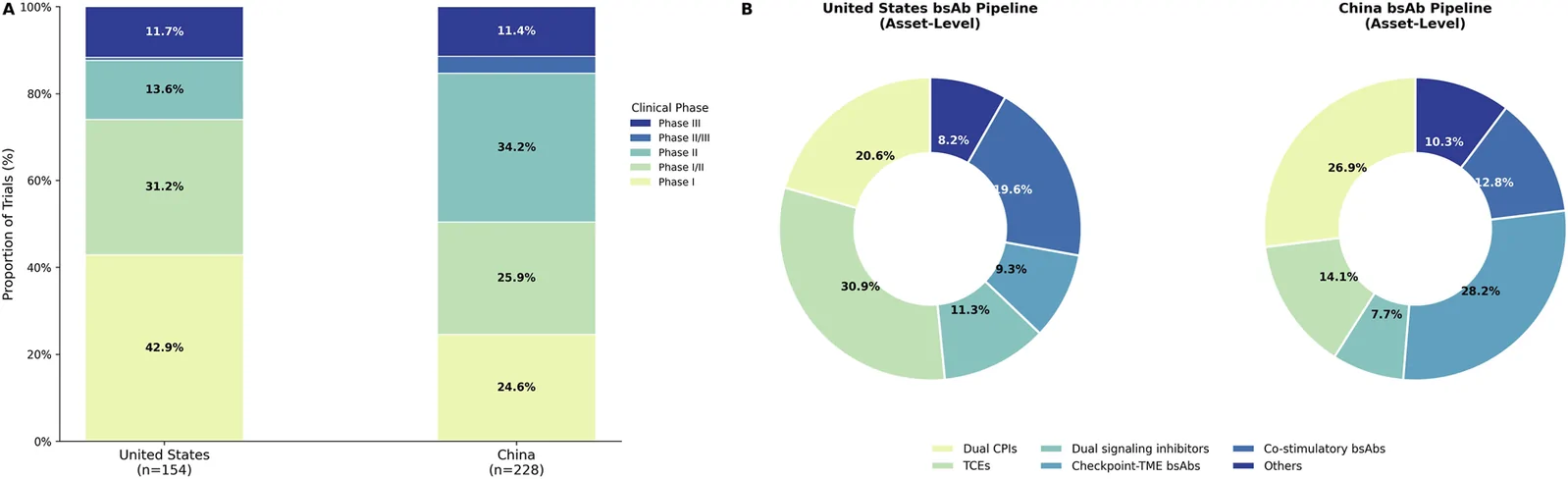
Divergent clinical development patterns between mainland China and the United States. **(A)** Phase distribution at the clinical-trial level among trials involving the United States (n = 154) or mainland China (n = 228). **(B)** Mechanism distribution at the unique-asset level among assets studied in the United States (n = 97) or mainland China (n = 78). Multi-regional trials and assets studied in both countries could contribute to both groups. “Others” includes tumor-targeted immunomodulators and other exploratory targets with low pipeline frequency. These descriptive, participation-based groups differ from the mutually exclusive US-only and mainland-China-only subsets used for inferential comparisons in Section 3.3; subset definitions and sample sizes are provided in Supplementary Table S3.

Despite this shared dominance, the two nations pursue divergent clinical and strategic trajectories. Trials conducted in mainland China show rapid progression into late-stage evaluation, featuring over 110 active trials currently in Phase II and pivotal Phase III settings (Figure 6A; Table 1). Mechanistically, these trials are concentrated in well-validated target combinations—most notably Checkpoint-TME bsAbs (e.g., PD-(L)1 × VEGF) and Dual CPIs (e.g., PD-(L)1 × CTLA-4) (Figure 6B). In contrast, the US pipeline is heavily skewed toward early-stage exploration. Approximately 74% (114/154) of US-participating trials remain confined to Phase I or Phase I/II first-in-human studies (Figure 6A). Rather than focusing on validated target combinations, the US pipeline maintains a diversified emphasis on biologically novel, higher-risk modalities (Figure 6B). These include the development of complex TCEs, macrophage-directed innate immune modulators, and inhibitors targeting novel bypass signaling pathways.

Among mutually exclusive subsets—trials conducted in the United States but not mainland China (n = 113) and trials conducted in mainland China but not the United States (n = 187)—phase distribution differed significantly (χ^2^ = 32.17, df = 1, P < 0.001; Cramér’s V = 0.33). Mechanistic composition also differed, in an analysis restricted to the six defined mechanistic classes (n = 107 and n = 182, respectively; χ^2^ = 89.99, df = 5, Monte Carlo P < 0.001; Cramér’s V = 0.56; Supplementary Table S3).

### 3.4 Sponsor distribution and trial methodology

A distinct division of labor is evident among sponsor types (Supplementary Figure S1). Emerging biotechs (non-Top 20 industry sponsors) drive pipeline volume, initiating 61.2% (229/374) of all trials, with >70% of their efforts confined to Phase I or I/II. In contrast, Top 20 Pharma leads late-stage execution, exhibiting the highest intra-group proportion of Phase III trials (16.0%). Academic efforts are predominantly (73.5%) Phase II investigator-initiated trials (IITs).

Phase distribution differed significantly across the three principal sponsor categories (n = 359; χ^2^ = 50.01, df = 2, P < 0.001; Cramér’s V = 0.37; Supplementary Table S3).

Target selection further delineates these sponsors (Table 1). Top 20 Pharma preferentially develops precision, resistance-driven dual signaling inhibitors (e.g., the EGFR × c-MET bsAb amivantamab). Conversely, biotechs predominantly advance combinatorial immunomodulatory mechanisms. While globally concentrated in early phases, this immunomodulatory strategy has been notably propelled into late-stage validation by Chinese biotechs (e.g., the PD-(L)1 × VEGF bsAb ivonescimab).

Methodologically, approximately 30% of the active pipeline employs seamless trial designs (e.g., Phase I/II or Phase II/III adaptive transitions). Concurrently, late-stage validation increasingly relies on MRCTs, particularly in Top 20 Pharma-partnered programs.

## 4 Discussion

The clinical development of bsAbs in NSCLC represents a significant advance in oncological pharmacotherapy. Historically, durable responses to targeted therapies and ICIs have been limited by acquired resistance—including bypass signaling—and by primary or acquired immune resistance involving an adaptive, immunosuppressive TME, respectively (Sharma et al., 2017; Wu and Shih, 2018). Importantly, the therapeutic rationale for bsAbs extends beyond combining two monoclonal antibody targets into a single molecule. By enforcing simultaneous co-engagement of two epitopes on the same or neighboring cells, bsAbs achieve proximity-driven pharmacological effects such as obligate receptor cross-linking, spatially restricted immune activation, and enhanced avidity for tumor cells co-expressing both targets. These effects are mechanistically unattainable through co-administration of two separate monospecific antibodies (Klein et al., 2024).

As dual-signaling bsAbs enter late-stage development, the key clinical question is when they should be used. Amivantamab demonstrated clinical activity in EGFR exon 20 insertion-mutated NSCLC in the post-platinum setting and subsequently in combination with chemotherapy in the first-line setting (Park et al., 2021; Zhou et al., 2023). In MARIPOSA, first-line amivantamab plus lazertinib improved PFS and OS over osimertinib in common EGFR-mutant NSCLC (Cho et al., 2024; Yang et al., 2025), but grade ≥3 adverse events occurred in 80% versus 52% of patients (Yang et al., 2025). MARIPOSA-2 demonstrated improved PFS with amivantamab plus chemotherapy after progression on osimertinib (Passaro et al., 2024). However, these trials did not compare the two treatment sequences. Whether EGFR-MET blockade should be introduced first-line or reserved for acquired resistance therefore remains unresolved.

Checkpoint-TME bsAbs are likewise being studied across treatment lines. HARMONi-2 showed longer PFS with ivonescimab than pembrolizumab monotherapy in untreated PD-L1-positive NSCLC (Xiong et al., 2025). Because pembrolizumab monotherapy was the sole comparator, ivonescimab’s position relative to chemoimmunotherapy and other first-line options remains uncertain. IBI363 is being developed mainly after progression on PD-(L)1 therapy (Table 1), representing a distinct post-resistance strategy. Future trials should determine whether checkpoint-TME bsAbs are better used to intensify initial immunotherapy or overcome established resistance, using control regimens appropriate to each treatment line.

Another prominent mechanistic trajectory is the expansion of TCEs. This approach, historically successful in hematologic malignancies, has increasingly been applied to solid tumors. Our analysis showed a growing number of TCE trials, although most remained in early-stage development. TCE trials also had a higher termination rate than trials in all other mechanistic classes combined. However, nine of the 14 terminated TCE trials were coded as business decisions; the remainder were coded as poor enrollment (n = 2), other (n = 2), or unknown (n = 1) (Supplementary Table S3). This distribution indicates that the higher observed termination rate should not be interpreted as direct evidence of mechanism-level failure.

Several factors may nevertheless contribute to the difficulty of developing TCEs for solid tumors. Biologically, heterogeneous or insufficient target expression and an immunosuppressive tumor microenvironment may limit effective and durable tumor-cell engagement. From a safety perspective, cytokine release syndrome and on-target/off-tumor toxicity may narrow the therapeutic window and complicate dose optimization (Van De Donk and Zweegman, 2023). The clinical activity of the DLL3-directed TCE tarlatamab in small-cell lung cancer demonstrates the feasibility of T-cell redirection in a thoracic malignancy, while also illustrating the need for careful dose and toxicity management (Ahn et al., 2023). Commercial factors, including portfolio reprioritization and resource allocation, may also lead to trial discontinuation independently of a compound’s biological activity. Accordingly, the higher termination rate observed among TCE trials likely reflects a mixture of development-related and portfolio-level factors. Registry-recorded categories do not capture the reasoning behind individual decisions, and termination of a trial does not necessarily indicate failure of the therapeutic asset or the TCE mechanism.

Beyond mechanistic evolution, our analysis identifies distinct but potentially complementary development patterns associated with sponsor identity and geographic trial participation. In China, the clinical pipeline is driven by emerging biotechs and academic institutions focused on validated combinatorial approaches, most notably checkpoint-TME bsAbs (e.g., the PD-1 × VEGF bsAb ivonescimab) and dual CPIs (e.g., the PD-1 × CTLA-4 bsAb cadonilimab). This pattern is directly visible in the registration data: Chinese trials concentrate on a small number of validated target combinations and advance them rapidly into Phase II and Phase III settings. The clinical relevance of this development pattern is illustrated by HARMONi-2, in which ivonescimab significantly prolonged progression-free survival compared with pembrolizumab monotherapy in first-line PD-L1-positive NSCLC (Xiong et al., 2025). The reasons for this concentration, however, are not examined by the present dataset. One plausible explanation comes from the regulatory literature. The National Medical Products Administration (NMPA) has increasingly scrutinized redundant targets and emphasized evidence of benefit relative to existing standards of care (Zhou et al., 2017; Zhong et al., 2021). Under such conditions, using clinically validated target combinations would be expected to reduce the failure risk associated with novel target discovery. This interpretation is consistent with the distribution we observe. However, registration records do not capture sponsor decision-making. We therefore treat it as a hypothesis rather than a finding of this analysis. Nevertheless, this concentration on validated targets also raises concerns about pipeline redundancy. Multiple domestic developers are pursuing similar bsAb constructs with overlapping mechanisms. This may lead to resource inefficiency and diminishing returns (Huang et al., 2022).

Conversely, the US landscape is skewed toward early-stage trials of TCEs and innate immune modulators, a difference directly supported by our data (Section 3.3). The determinants of this orientation lie outside the registration record. Two explanations recur in the literature. First, the US venture-capital ecosystem has been described as disproportionately rewarding first-in-class biological breakthroughs (Kennedy et al., 2023). Second, the FDA’s Project Optimus initiative requires more extensive dose optimization during early development and may help explain why a larger share of US programs remained in early phases (Shah et al., 2021). Neither factor was directly examined in the present study, and we present them as plausible contributors rather than demonstrated causes. What our data establish is the resulting profile: a pipeline concentrated in earlier phases but distributed across a broader range of mechanisms.

To bridge these divergent regional paradigms and accelerate global drug availability, international collaboration and agile trial methodologies may be increasingly important. Seamless clinical trial designs, including Phase I/II adaptive transitions, may be particularly useful when combined with cross-border licensing and MRCTs. Recent agreements between Chinese biotechs and global sponsors, including the Summit Therapeutics–Akeso partnership for ivonescimab, illustrate how regionally developed assets can enter broader late-stage programs. Such arrangements may connect complementary strengths in patient access, mechanistic innovation, and global trial execution.

This study has several limitations. First, our analysis relied exclusively on the Trialtrove database. Although Trialtrove aggregates data from more than 30,000 global sources, reliance on a single proprietary registry has specific consequences for completeness and verifiability. Trials registered only on regional platforms not indexed by Trialtrove (for example, JRCT or CRIS) may have been missed, which may underrepresent certain regions. The curation and mechanistic annotation applied by the database provider are not fully transparent to end users, and any systematic bias in that curation would propagate into our dataset. This is one reason we re-reviewed every record manually rather than relying on the database’s own modality classification. Finally, because access is licensed, independent replication requires an institutional subscription. To mitigate these limitations, we report the full search and data-handling procedures (Supplementary Tables S1,S2). We also provide aggregated trial-level distributions and analytic populations for the principal descriptive and inferential analyses (Supplementary Table S3), thereby improving the traceability of the reported results.

Second, the mechanism-based classification framework used in this study was a study-specific taxonomy developed to reflect the observed structure of the NSCLC bispecific antibody pipeline, rather than a universally standardized classification system. Criteria were pre-specified, assignment was determined by the identity of the engaged targets, and classification decisions were independently reviewed by two investigators (Section 2.3). Nevertheless, the definition of category boundaries necessarily involved some judgment in borderline cases, particularly when a target had overlapping biological functions. For example, CD47 may be viewed both as an innate immune checkpoint and as a tumor-directed immunomodulatory target, whereas cytokines such as IL-2 can modulate the tumor microenvironment while simultaneously delivering an agonistic signal. Such constructs could reasonably be assigned to more than one category, and alternative taxonomies might therefore produce modest differences in category sizes. The broad developmental patterns identified in this study are unlikely to be materially altered by reassignment of a small number of borderline assets. These patterns include the predominantly early-phase status of TCEs, the more rapid progression of checkpoint-TME bispecific antibodies into later-stage development, and the differences between the US and Chinese pipelines. Nevertheless, the exact category proportions should be interpreted as approximate rather than definitive.

Third, bispecific antibody-drug conjugates (bsADCs) were intentionally excluded to independently evaluate the intrinsic properties of unconjugated bispecific formats; however, this exclusion limits the comprehensiveness of the overall landscape assessment, particularly as bsADCs represent a rapidly emerging modality. Fourth, as a landscape analysis based on trial registration data, this study was not designed to evaluate the methodological quality of individual trials or to assess clinical efficacy outcomes. Fifth, geographic distribution was analyzed based on registered study-site countries. Country participation does not identify the origin of an asset, the nationality of the sponsor, or exclusive development by a single country. Multi-regional trials contributed to the counts of several countries, so country-level descriptive counts were not mutually exclusive. The regional differences we report should therefore be interpreted as differences in participation patterns rather than in development attribution. To avoid overlap in the inferential comparisons, comparisons between the United States and mainland China used mutually exclusive subsets (Section 3.3; Supplementary Table S3). In addition, 12 trials lacking geographic information were excluded from this analysis. Finally, given the rapidly evolving nature of the bsAb field, new trials initiated or pivotal data disclosed after our data cutoff of February 2026 could not be captured in the present analysis. Furthermore, this study did not conduct a systematic quantitative analysis of combination therapy strategies across the pipeline, which warrants dedicated investigation in future research.

## 5 Conclusion and perspectives

**FIGURE 7:**
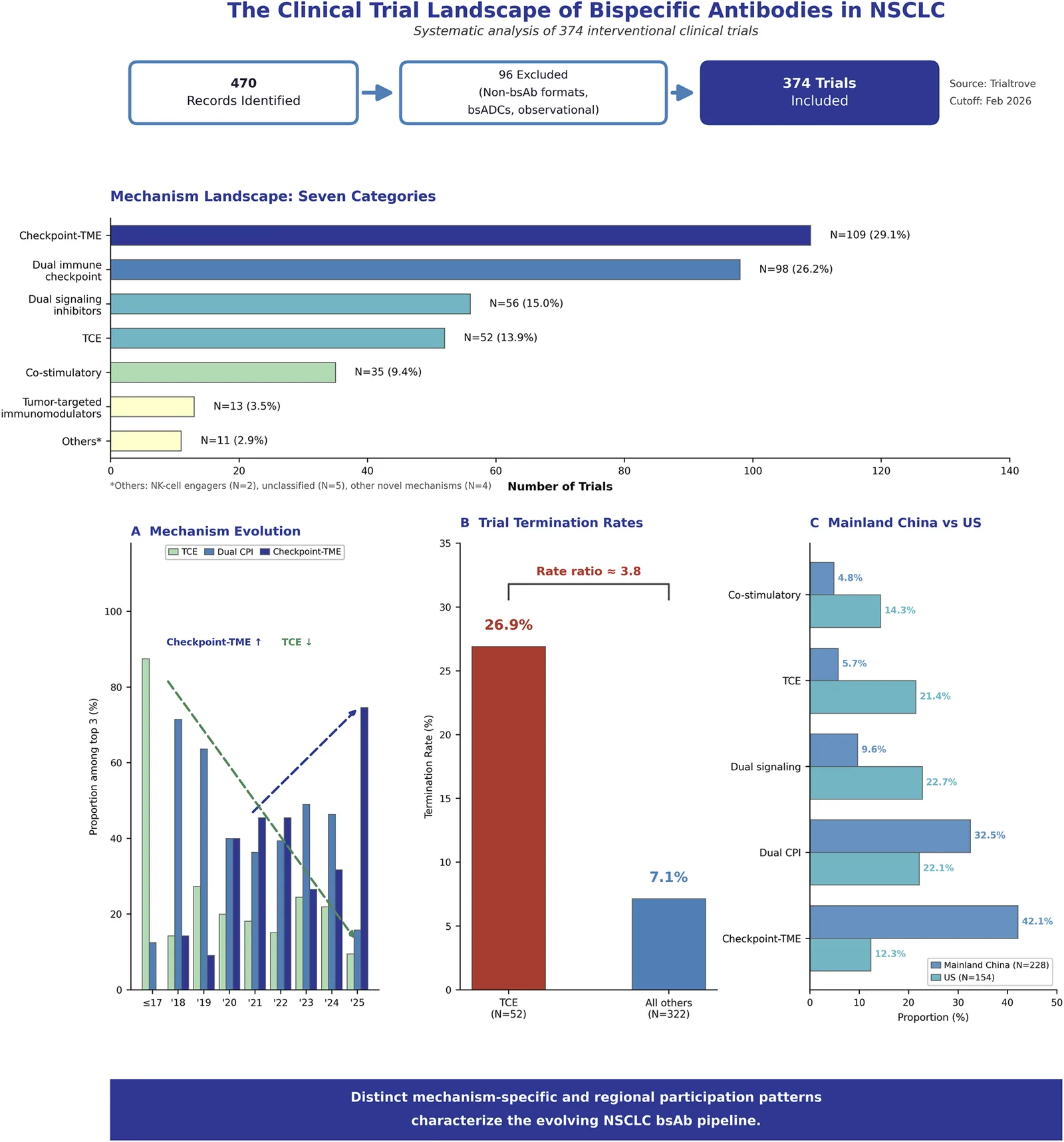
Visual summary of the clinical trial landscape of bispecific antibodies in NSCLC. The unit of analysis was the individual clinical trial. The top panel illustrates trial selection, and the horizontal bar chart shows the distribution of trials across seven mechanistic categories (N = 374). **(A)** Temporal evolution of the three dominant mechanistic classes; the denominator in each period comprised trials in the three displayed classes. **(B)** Trial termination rates comparing TCEs (n = 52) with all other mechanistic classes (n = 322). **(C)** Participation-based mechanism distributions among trials involving mainland China (n = 228) or the United States (n = 154); a multi-regional trial involving both countries could contribute to both groups. These descriptive groups differ from the mutually exclusive country subsets used for inferential comparisons in Section 3.3; definitions and sample sizes are provided in Supplementary Table S3.

In summary, our analysis of 374 clinical trials identifies a decisive shift in the NSCLC bsAb pipeline toward rationally engineered constructs designed to overcome single-agent resistance (Figure 7). This evolution follows a stratified pattern of clinical maturation: dual-signaling inhibitors, checkpoint-TME modulators, and dual CPIs have advanced into late-stage pivotal validation, while TCEs and conditional co-stimulatory bsAbs drive a rapidly expanding wave of early-stage exploration. This trajectory is reinforced by a complementary geographic divergence that balances high-risk innovation with rapid clinical execution, further accelerated by seamless trial methodologies and cross-border collaborations.

The integration of advanced bsAbs into standard NSCLC care will ultimately depend on mature overall survival data from ongoing Phase III trials. Concurrently, realizing their full clinical potential requires addressing key translational challenges, including the identification of robust predictive biomarkers, management of modality-specific toxicities (e.g., cytokine release syndrome), and optimization of treatment sequencing. Beyond these unconjugated modalities, the field is already advancing toward bispecific antibody-drug conjugates (bsADCs). Although excluded from the present analysis to maintain methodological focus, bsADCs represent a convergence of precise dual-targeting and potent cytotoxicity, and are emerging as a significant trajectory for next-generation precision therapeutics.

## Data Availability

The data analyzed in this study is subject to the following licenses/restrictions: The clinical trial data analyzed in this study were retrieved from the proprietary Trialtrove database (Citeline) under institutional license and therefore are not redistributed as a record-level extract. Aggregated data supporting the principal descriptive and inferential analyses are provided in Supplementary Table S3. Requests to access these datasets should be directed to ZDL, bs0786@shutcm.edu.cn.
