# Supplementary material for: The clinical trial landscape of bispecific antibodies in non-small cell lung cancer: mechanistic trends and developmental strategies
Source: Front Pharmacol. 2026 Sep 3;17:1901850. doi: 10.3389/fphar.2026.1901850 (PMC13581824; doi:10.3389/fphar.2026.1901850)
Supplement: Supplementary file 1 [file Image1.pdf]

Supplementary Material

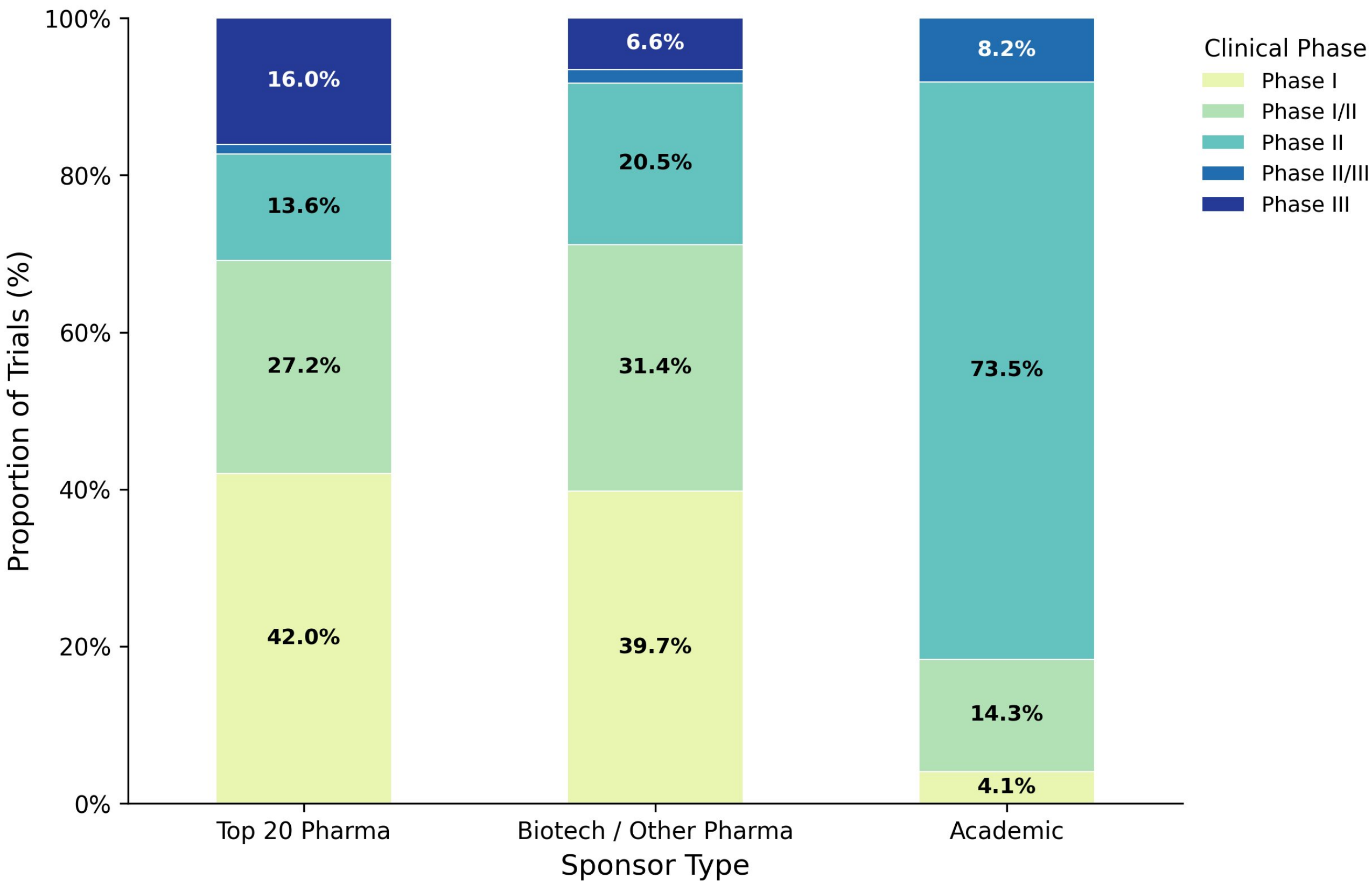

Supplementary Figure S1. Clinical phase distribution by primary sponsor category. The unit of analysis was the individual clinical trial. When more than one sponsor category was listed, a single primary category was assigned using the precedence rule in Supplementary Table S2.
