# Supplementary material for: The clinical trial landscape of bispecific antibodies in non-small cell lung cancer: mechanistic trends and developmental strategies
Source: Front Pharmacol. 2026 Sep 3;17:1901850. doi: 10.3389/fphar.2026.1901850 (PMC13581824; doi:10.3389/fphar.2026.1901850)
Supplement: Supplementary file 2 [file Table1.docx]

Supplementary Material

**Supplementary Table S1**

**Supplementary Table S1.** Complete search fields, hierarchical terms, Boolean operators, date restriction, and number of records retrieved.

| **Search component** | **Database field** | **Search term / specification** | **Boolean relationship** |
| --- | --- | --- | --- |
| **Database** | — | Trialtrove | — |
| **Search date / cutoff** | — | February 8, 2026 | — |
| **Drug-type block 1** | Drug Type | Biological > Protein > Antibody > Bispecific antibody | OR |
| **Drug-type block 2** | Drug Type | Biological > Protein > Antibody > Cell engager, bispecific | Combined with drug-type block 1 |
| **Disease block** | Disease | Oncology: Lung, Non-Small Cell | AND |
| **Date block 1** | Actual Start Date | Up to 2026/02/08 | OR |
| **Date block 2** | Anticipated Start Date | Up to 2026/02/08 | Combined with date block 1 |
| **Overall Boolean logic** | — | (Drug-type block 1 OR drug-type block 2) AND disease block AND (date block 1 OR date block 2) | — |
| **Records retrieved** | — | 470 trials | — |
| **Records included** | — | 374 trials (96 excluded during manual screening; see Figure 1) | — |

**Exact exported query**

[(Drug Type is Biological > Protein > Antibody > Bispecific antibody) OR (Drug Type is Biological > Protein > Antibody > Cell engager, bispecific)] AND (Disease is Oncology: Lung, Non-Small Cell) AND [(Actual Start Date is to 2026/02/08) OR (Anticipated Start Date is to 2026/02/08)]
