# Supplementary material for: The clinical trial landscape of bispecific antibodies in non-small cell lung cancer: mechanistic trends and developmental strategies
Source: Front Pharmacol. 2026 Sep 3;17:1901850. doi: 10.3389/fphar.2026.1901850 (PMC13581824; doi:10.3389/fphar.2026.1901850)
Supplement: Supplementary file 3 [file Table2.docx]

Supplementary Material

**Supplementary Table S2**

**Supplementary Table S2.** Extracted variables and their handling, the precedence rule applied to multi-category sponsor records, and the predefined framework used to assign mechanistic categories.

**Part A. Extracted variables**

| **Variable** | **Definition** | **Handling of missing or multi-valued entries** |
| --- | --- | --- |
| **Trial identifier** | Primary registry number (NCT, ChiCTR, EudraCT, or equivalent) | Records without a valid registration number were excluded during manual screening |
| **Trial initiation year** | Year component of the registered study start date | Complete for all 374 included trials |
| **Trial phase** | Registered phase: I, I/II, II, II/III, III, or IV | Complete for all 374 included trials |
| **Operational status** | Open, Planned, Closed, Completed, Temporarily Closed, or Terminated, as recorded at extraction | Complete for all 374 included trials |
| **Primary sponsor type** | Trialtrove sponsor categorization | Where several sponsor types were listed, one category was assigned by the precedence rule in Part B |
| **Study locations** | Registered study-site countries | 12 trials lacked location data and were excluded from the geographic analysis only (denominator 362); multi-regional trials contribute to every participating country |
| **Investigational drug** | Agent name, standardized across synonyms and development codes | Standardized names were used for deduplication to 170 unique assets |
| **Molecular targets** | The antigens engaged by each arm of the construct | Trials with undisclosed targets were excluded during manual screening |
| **Mechanistic category** | One of the seven categories defined in Part C | Assets with information insufficient to identify both targets were assigned to “Other targets” rather than excluded |

**Part B. Precedence rule for sponsor type**

Trialtrove may list more than one sponsor type for a single trial. To obtain one mutually exclusive category per trial, the following precedence order was applied, taking the first category present:

Government → Cooperative Group → Industry, Top 20 Pharma → Industry, all other pharma → Academic

Trials matching none of these categories were assigned to “Others”. This rule yields the distribution reported in Figure 2C: industry, all other pharma 229; industry, Top 20 Pharma 81; academic 49; government 6; cooperative group 4; others 5 (total 374).

**Part C. Mechanistic classification codebook**

Criteria were defined a priori, before data extraction, and applied unchanged throughout. Classification was performed at the unique-asset level; all trials evaluating the same asset share its category. Assignment was determined by the identity of the engaged targets rather than by narrative descriptions of therapeutic intent.

| **Category** | **Defining feature (target identity)** | **Representative target pairs** |
| --- | --- | --- |
| **Dual signaling inhibitors** | Neither arm is immune-directed; both engage oncogenic signaling or angiogenic pathways | EGFR × c-MET; EGFR × HER3; HER2 × HER3; Ang-2 × VEGF |
| **T-cell engagers (TCEs)** | One arm engages a T-cell activating receptor (CD3) | EGFR × CD3; CLDN6 × CD3; EpCAM × CD3; CEA × CD3 |
| **Dual immune checkpoint inhibitors (Dual CPIs)** | Both arms engage inhibitory immune checkpoints | PD-1 × CTLA-4; PD-1 × TIGIT; PD-1 × LAG-3; PD-L1 × TIM-3 |
| **Checkpoint-TME bsAbs** | One arm engages an inhibitory checkpoint; the other engages a non-checkpoint modulator of the tumor microenvironment — a soluble suppressive factor, an immunostimulatory cytokine, or an innate immune checkpoint | PD-1 × VEGF; PD-L1 × TGF-β; PD-1 × IL-2; PD-L1 × CD47 |
| **Co-stimulatory bsAbs** | One arm engages an agonistic T-cell co-stimulatory receptor (4-1BB, OX40, ICOS, CD27, CD28), irrespective of the partner arm | PD-L1 × 4-1BB; CEA × 4-1BB; EGFR × CD28; PD-L1 × OX40 |
| **Tumor-targeted immunomodulators** | One arm engages a tumor-associated antigen; the other engages an immune checkpoint or an immunomodulatory receptor acting principally on antigen-presenting or innate immune cells (CD40, CD47) | HER2 × PD-1; CLDN18.2 × CD47; EpCAM × CD40; MSLN × CD47 |
| **Other targets** | Unconventional or low-frequency mechanisms, including innate and non-conventional cell engagers, and assets for which both targets could not be identified | EGFR × CD16A; EGFR × Vγ9Vδ2 |

**Part D. Precedence order for constructs satisfying more than one category**

Some constructs meet the defining feature of more than one category — for example, PD-1 × 4-1BB engages both an inhibitory checkpoint and a co-stimulatory receptor. A fixed precedence order was therefore applied:

| **Order** | **Criterion** | **Rationale** |
| --- | --- | --- |
| **1** | Engagement of a T-cell activating receptor (CD3) takes precedence over all other features | Introduction of CD3 determines the pharmacology, toxicity profile, and development pathway of the construct; the partner arm functions principally as a targeting moiety |
| **2** | Engagement of a T-cell co-stimulatory receptor takes precedence over checkpoint or tumor-antigen features | Agonistic co-stimulation governs the dose-limiting toxicity and conditional-activation strategy of the construct |
| **3** | No further precedence is required; remaining constructs are assigned directly by the definitions in Part C | Once criteria 1 and 2 have been applied, the remaining categories no longer overlap, and the target pair alone determines the assignment |

Because the rule operates on target identity rather than on investigator judgment, assignment is reproducible from the target pair itself. Classification decisions were independently reviewed by two investigators (Z.L. and X.W.). Discrepancies were resolved by discussion, and cases that remained unresolved were adjudicated by a third senior investigator (Z.-D.L.).

**Note. Bispecific antibody–drug conjugates were excluded from classification by design. A small number of constructs lie close to category boundaries, and alternative classification schemes would yield somewhat different category sizes; this is discussed in the Limitations section of the main text.**
