# Supplementary material for: The clinical trial landscape of bispecific antibodies in non-small cell lung cancer: mechanistic trends and developmental strategies
Source: Front Pharmacol. 2026 Sep 3;17:1901850. doi: 10.3389/fphar.2026.1901850 (PMC13581824; doi:10.3389/fphar.2026.1901850)
Supplement: Supplementary file 4 [file Table3.docx]

Supplementary Material

**Supplementary Table S3**

**Supplementary Table S3. Aggregated trial-level distributions and analytic populations used in the descriptive and inferential analyses.** Counts are presented by mechanistic class, clinical phase, sponsor category, operational status, initiation year, and country.

**S3a. Mechanistic class by clinical phase**

Unit of analysis: clinical trials. Denominator: all 374 included trials. These are the five-level phase categories retained for descriptive analyses.

| **Mechanistic class** | **Phase I** | **Phase I/II** | **Phase II** | **Phase II/III** | **Phase III** | **Total** |
| --- | --- | --- | --- | --- | --- | --- |
| **Dual signaling inhibitors** | 19 | 13 | 17 | 2 | 5 | 56 |
| **T-cell engagers** | 35 | 15 | 2 | 0 | 0 | 52 |
| **Dual CPIs** | 22 | 28 | 36 | 3 | 9 | 98 |
| **Checkpoint-TME bsAbs** | 20 | 30 | 42 | 4 | 13 | 109 |
| **Co-stimulatory bsAbs** | 19 | 11 | 4 | 0 | 1 | 35 |
| **Tumor-targeted immunomodulators** | 10 | 2 | 1 | 0 | 0 | 13 |
| **Other targets** | 4 | 6 | 1 | 0 | 0 | 11 |
| **Total** | **129** | **105** | **103** | **9** | **28** | **374** |

**S3b. Sponsor category by clinical phase**

Unit of analysis: clinical trials. Denominator: all 374 included trials. Sponsor category was assigned by the precedence rule given in Supplementary Table S2, Part B.

| **Sponsor category** | **Phase I** | **Phase I/II** | **Phase II** | **Phase II/III** | **Phase III** | **Total** |
| --- | --- | --- | --- | --- | --- | --- |
| **Industry, Top 20 Pharma** | 34 | 22 | 11 | 1 | 13 | 81 |
| **Industry, all other pharma** | 91 | 72 | 47 | 4 | 15 | 229 |
| **Academic** | 2 | 7 | 36 | 4 | 0 | 49 |
| **Government** | 1 | 1 | 4 | 0 | 0 | 6 |
| **Cooperative Group** | 0 | 1 | 3 | 0 | 0 | 4 |
| **Others** | 1 | 2 | 2 | 0 | 0 | 5 |
| **Total** | **129** | **105** | **103** | **9** | **28** | **374** |

**S3c. Mechanistic class by operational status**

Unit of analysis: clinical trials. Denominator: all 374 included trials. Status was recorded at the time of data extraction (February 8, 2026).

| **Mechanistic class** | **Open** | **Planned** | **Closed** | **Completed** | **Temporarily**  **closed** | **Terminated** | **Total** |
| --- | --- | --- | --- | --- | --- | --- | --- |
| **Dual signaling inhibitors** | 19 | 6 | 12 | 16 | 0 | 3 | 56 |
| **T-cell engagers** | 24 | 3 | 5 | 6 | 0 | 14 | 52 |
| **Dual CPIs** | 46 | 16 | 8 | 19 | 0 | 9 | 98 |
| **Checkpoint-TME bsAbs** | 56 | 31 | 6 | 13 | 0 | 3 | 109 |
| **Co-stimulatory bsAbs** | 15 | 0 | 6 | 10 | 0 | 4 | 35 |
| **Tumor-targeted immunomodulators** | 8 | 0 | 0 | 3 | 1 | 1 | 13 |
| **Other targets** | 6 | 0 | 1 | 1 | 0 | 3 | 11 |
| **Total** | **174** | **56** | **38** | **68** | **1** | **37** | **374** |

**S3d. Trials initiated per year**

Unit of analysis: clinical trials. Denominator: all 374 included trials. Data for 2026 are incomplete, as the search cutoff was February 8, 2026.

| **Year** | **Trials** | **Year** | **Trials** | **Year** | **Trials** | **Year** | **Trials** |
| --- | --- | --- | --- | --- | --- | --- | --- |
| **2008** | 1 | 2016 | 6 | 2020 | 27 | 2024 | 57 |
| **2012** | 1 | 2017 | 4 | 2021 | 38 | 2025 | 78 |
| **2014** | 1 | 2018 | 17 | 2022 | 51 | 2026 | 3 |
| **2015** | **3** | **2019** | **19** | **2023** | **68** |  |  |

**S3e. United States and mainland China participation (descriptive analyses)**

Unit of analysis: country–trial participations. Denominator: the 362 trials with available location data. Multi-regional trials contribute to every participating country, so the two rows are not mutually exclusive and do not sum to 362: 41 trials were sited in both the United States and mainland China. These participation-based counts are those shown in Figures 5, 6, and 7. “Mainland China” excludes Hong Kong SAR and Taiwan, which are counted separately.

| **Country** | **Phase I** | **Phase I/II** | **Phase II** | **Phase II/III** | **Phase III** | **Total** |
| --- | --- | --- | --- | --- | --- | --- |
| **United States (n = 154)** | 66 | 48 | 21 | 1 | 18 | 154 |
| **Mainland China (n = 228)** | 56 | 59 | 78 | 9 | 26 | 228 |

| **Country** | **Dual**  **signaling** | **TCEs** | **Dual**  **CPIs** | **Checkpoint-**  **TME** | **Co-**  **stimulatory** | **Tumor-**  **targeted** | **Other** | **Total** |
| --- | --- | --- | --- | --- | --- | --- | --- | --- |
| **United States (n = 154)** | 35 | 33 | 34 | 19 | 22 | 5 | 6 | 154 |
| **Mainland China (n = 228)** | 22 | 13 | 74 | 96 | 11 | 7 | 5 | 228 |

**S3f. Mutually exclusive country subsets (inferential analyses)**

Unit of analysis: clinical trials. Because participation-based country counts are not mutually exclusive, inferential comparisons were restricted to trials involving exactly one of the two focal countries. Trials involving both countries, neither country, or lacking location data were excluded from these comparisons.

| **Subset** | **Phase I** | **Phase I/II** | **Phase II** | **Phase II/III** | **Phase III** | **Total** |
| --- | --- | --- | --- | --- | --- | --- |
| **US participation without mainland China participation (n = 113)** | 59 | 37 | 15 | 0 | 2 | 113 |
| **Mainland China participation without US participation (n = 187)** | 49 | 48 | 72 | 8 | 10 | 187 |

| **Subset** | **Dual**  **signaling** | **TCEs** | **Dual**  **CPIs** | **Checkpoint-**  **TME** | **Co-**  **stimulatory** | **Tumor-**  **targeted** | **Other** | **Total** |
| --- | --- | --- | --- | --- | --- | --- | --- | --- |
| **US participation without mainland China participation (n = 113)** | 24 | 31 | 18 | 8 | 21 | 5 | 6 | 113 |
| **Mainland China participation without US participation (n = 187)** | 11 | 11 | 58 | 85 | 10 | 7 | 5 | 187 |

**S3g. Summary of statistical comparisons**

Expected cell counts were examined for each contingency table. When the assumptions of the Pearson chi-square approximation were not met, phase categories were collapsed or a Monte Carlo-estimated P value was used, as indicated.

| **Comparison** | **Analytic population** | **Test** | **Result** | **Rationale for test selection** |
| --- | --- | --- | --- | --- |
| **Phase (early vs Phase II or later) by country** | US participation without mainland China participation (n = 113) vs mainland China participation without US participation (n = 187) | Pearson chi-square (2 x 2, with continuity correction) | chi-square = 32.17, df = 1, P < 0.001; Cramer's V = 0.33 | All expected counts exceeded 5 (smallest 40.3); phase was collapsed to two levels to give a single interpretable contrast, and the continuity correction specified in Methods 2.4 was applied to this 2 x 2 table |
| **Mechanistic class by country** | US participation without mainland China participation (107 classified) vs mainland China participation without US participation (182 classified) | Pearson chi-square with Monte Carlo-estimated P value (2 x 6) | chi-square = 89.99, df = 5; Monte Carlo P < 0.001; Cramer's V = 0.56 | Smallest expected count was 4.4, below the conventional threshold, so the asymptotic approximation was not relied upon |
| **Phase (early vs Phase II or later) by sponsor category** | Top 20 pharma, other industry, and academic sponsors (n = 359) | Pearson chi-square (3 x 2) | chi-square = 50.01, df = 2, P < 0.001; Cramer's V = 0.37 | All expected counts exceeded 5 (smallest 17.9) after restriction to the three main sponsor categories |
| **Termination by mechanistic class** | Six defined mechanistic classes; "Other targets" excluded (n = 363) | Pearson chi-square with Monte Carlo-estimated P value (6 x 2) | chi-square = 25.78, df = 5; Monte Carlo P < 0.001; Cramer's V = 0.27 | Smallest expected count was 1.2, so a Monte Carlo-estimated P value was used instead of the asymptotic approximation |
| **Termination, TCEs vs all other classes** | All included trials (n = 374) | Fisher's exact test (2 x 2) | 26.9% (14/52) vs 7.1% (23/322); OR 4.79, 95% CI 2.27-10.09; Fisher's exact P < 0.001 | A targeted exact test was used for this single pre-specified contrast, so that an odds ratio with an exact 95% confidence interval could be reported alongside the P value. |

**S3h. Reasons recorded for terminated T-cell engager trials**

Unit of analysis: clinical trials. Denominator: the 14 terminated T-cell engager trials in S3c. Reasons are those recorded in Trialtrove at the time of data extraction; the database records a reason category rather than the underlying decision.

| **Recorded reason for termination** | **Trials** |
| --- | --- |
| **Business or strategic decision** | 9 |
| **Poor enrollment** | 2 |
| **Other** | 2 |
| **Not recorded** | 1 |
| **Total** | **14** |

Note. Counts in S3a-S3d and S3f are mutually exclusive and sum to the stated denominators; counts in S3e are participation-based and are not mutually exclusive. Twelve trials lacked location data and were excluded from S3e and S3f only. The heterogeneous "Other targets" category was retained in descriptive tables but excluded from inferential comparisons of mechanistic classes because it does not represent a single biologically coherent category. Inferential comparisons by sponsor category were restricted to Top 20 pharmaceutical companies, other industry sponsors, and academic sponsors; government, cooperative-group, and other sponsors were retained descriptively but excluded from formal testing because of small cell counts.

**Data availability note.** Trialtrove is a proprietary database, and record-level extracts cannot be redistributed. Aggregated counts are provided here to support transparency and verification of the reported analyses.
